# Temperature-controlled Laminar Airflow (TLA) in symptomatic severe asthma – a post hoc analysis of severe exacerbations, quality of life and health economics

**DOI:** 10.1186/s12890-022-02205-6

**Published:** 2022-11-09

**Authors:** A. J. Chauhan, G. Eriksson, W. Storrar, T. Brown, S. Peterson, F. Radner, L. G. D’Cruz, P. Miller, L. Bjermer

**Affiliations:** 1 Portsmouth Hospitals University NHS Trust and University of Portsmouth, Portsmouth, UK; 2 Department of Respiratory Medicine and Allergology, University Hospital, Lund, Sweden; 3 StatMind Statistical and Mathematical Modelling, Innovation and Design AB, Lund, Sweden; 4 Miller Economics Ltd. BIOHUB, Alderley Park, Alderley Edge, UK

**Keywords:** Temperature-controlled laminar airflow, Severe asthma, Severe exacerbations, Quality of life, Health economics

## Abstract

**Purpose:**

Uncontrolled severe asthma constitutes a major economic burden to society. Add-ons to standard inhaled treatments include inexpensive oral corticosteroids and expensive biologics. Nocturnal treatment with Temperature-controlled Laminar Airflow (TLA; Airsonett®) could be an effective, safe and cheaper alternative. The potential of TLA in reducing severe asthma exacerbations was addressed in a recent randomised placebo-controlled trial (RCT) in patients with severe asthma (Global Initiative for Asthma (GINA) step 4/5), but the results were inconclusive. We re-analysed the RCT with severe exacerbations stratified by the level of baseline asthma symptoms and Quality of Life.

**Methods:**

More uncontrolled patients, defined by Asthma Control Questionnaire 7 (ACQ7) > 3, EuroQoL 5-Dimension Questionnaire Visual Analogue Scale (EQ5D-VAS) ≤ 65 and Asthma Quality of Life Questionnaire (AQLQ) ≤ 4 were selected for re-analysis. The rates of severe asthma exacerbations, changes in QoL and health-economics were analysed and compared between TLA and placebo.

**Results:**

The study population included 226 patients (113 TLA / 113 placebo.) The rates of severe asthma exacerbations were reduced by 33, 31 and 25% (*p* = 0.083, 0.073, 0.180) for TLA compared to placebo, dependent on selected control measures (ACQ7, EQ5D-VAS, AQLQ, respectively). For patients with less control defined by AQLQ≤4, the difference in mean AQLQ_0-12M_ between TLA and placebo was 0.31, 0.33, 0.26 (*p* = 0.085, 0.034, 0.150), dependent on selected covariate (AQLQ, EQ5D-VAS, ACQ7, respectively). For patients with poor control defined by ACQ7 > 3, the difference in EQ5D-5 L utility scores between TLA and placebo was significant at 9 and 12 months with a cost-effective ICER. The results from the original study did not demonstrate these differences.

**Conclusion:**

This post hoc analysis demonstrated an effect of TLA over placebo on severe exacerbations, asthma control and health economics in a subgroup of patients with more symptomatic severe allergic asthma. The results are consistent with the present recommendations for TLA. However, these differences were not demonstrated in the full study. Several explanations for the different outcomes have been outlined, which should be addressed in future studies.

**Funding:**

NIHR Health Technology Assessment Programme and Portsmouth Hospitals NHS Trust.

## Background

Symptomatic severe asthma constitutes a major economic burden to society and the prognosis is that costs will continue to grow [1, 2]. Treatment alternatives include high-dose inhaled corticosteroids (ICS) plus a bronchodilator (e.g. long-acting β2-agonists and/or muscarinic antagonists) plus systemic corticosteroids, and treatments with biologics [3–5], according to Global Initiative for Asthma (GINA) step 4/5 [6]. Another add-on treatment option in symptomatic severe allergic asthma is nocturnal temperature-controlled laminar airflow (TLA). TLA reduces exposure to allergen and irritant particles during sleep by delivering filtered and slightly cooled air to the breathing zone of the patient [7, 8]. TLA improves the quality of life and decreases inflammatory markers [9, 10]. In addition, cost-effectiveness [11] has been demonstrated for TLA at ranges consistent with the National Institute for Health and Care Excellence (NICE) standards [12]. The TLA documentation has led to recommendations for symptomatic severe asthma in the Scottish and Swedish guidelines [13, 14].

In the double-blind 4A study by Boyle et al. [9], patients with uncontrolled atopic asthma (GINA step 2–4) were treated with TLA or placebo for 1 year, and a significant difference in the Asthma Quality of Life Questionnaire (AQLQ) responder rate was observed. In a predefined sub-analysis this effect was greater in patients with more severe asthma, and a significant effect was observed for total AQLQ already after 3 months in the subgroup of patients with Asthma Control Test (ACT) score < 18 at baseline and GINA step 4 [15]. This study was not designed to evaluate the rate of severe asthma exacerbations, but a non-significant difference in severe exacerbation rates (*p* = 0.07) between TLA and placebo was demonstrated in the subgroup of patients with symptomatic severe asthma, defined by ACT< 18 and GINA step 4.

To further evaluate the effects of TLA on asthma exacerbations, another one-year, double-blind, placebo-controlled study was designed, publicly known as the LASER study (for study protocol, see Storrar et al. [16]). Kapoor et al. [17] examined 240 severe asthma patients treated according to GINA step 4/5. The study found no significant effect on severe exacerbations (rate ratio = 0.92; *p* = 0.62) between TLA and placebo. The reason could be several-fold: 1) the finding in the study demonstrated a true lack of effect on severe exacerbations in the population studied; 2) the finding in the study failed to demonstrate an effect of TLA, because of certain short-comings e.g., inclusion of less symptomatic patents, a prominent placebo effect or any other reason.

Based on the 4A study results and in line with defined explorative analysis in the LASER protocol, we conducted the present post-hoc study in patients with more uncontrolled severe asthma at baseline. Our first objective was to examine the rate difference for severe exacerbations between TLA and placebo. The second objective was to evaluate Quality of Life (QoL) and cost-effectiveness.

## Methods

The current study is a post-hoc analysis of data from the LASER study (Kapoor et al., ClinicalTrials.gov NCT02813811) [17].

### Study design

The LASER study was a phase III multicentre, double-blind, placebo-controlled, parallel-group study in which patients were randomised to receive add-on treatment with TLA (Airsonett AIR4, Airsonett AB, Ängelholm; Sweden) or a placebo device for 1 year (randomisation 1:1). All participants were evaluated during the study at baseline and after 3, 6, 9 and 12 months, and via completion of a diary. The primary outcome was the frequency of severe asthma exacerbations occurring over 12 months, defined in accordance with the American Thoracic Society/European Respiratory Society (ATS/ERS) guidelines as a worsening of asthma requiring systemic corticosteroids, that is, ≥ 30 mg of prednisolone or equivalent daily (or a ≥ 50% increase in dose if maintenance 30 mg of prednisolone or above) for ≥3 days.

### Outcome measures

Our first objective was to examine the rate difference for severe exacerbations between TLA and placebo. Our second objective was to investigate QoL and health economics among patients with more uncontrolled asthma.

### Statistical analyses

For this post-hoc analysis, patients with more uncontrolled asthma were selected. Selections were made by splitting at the medians adjusted to the nearest even integer of ACQ7-, EQ5D-VAS- and AQLQ- scores. Cut-offs utilised were > 3 for ACQ7, ≤65 for EQ5D-VAS and ≤ 4 for AQLQ for exacerbations, ≤4 for AQLQ for QoL and > 3 for ACQ for the health economic evaluation. Analyses were performed separately in the sub-groups.

For rates of severe exacerbations, a negative binomial model was used with a factor for treatment. The average of AQLQ during the 12-month study was analysed in an ANOVA with AQLQ at baseline as a covariate. The same method was used for the domains of AQLQ. An MMRM (Mixed effect Model Repeat Measurement) approach was also tried but abandoned due to numerical convergence problems. For EQ5D-5 L utility data the MMRM was used with factors for treatment and visits and their interaction to display the time pattern. EQ5D-5 L at baseline was used as a covariate. The covariance matrix across visits was unstructured.

## Results

### Patients

The LASER study included 240 randomized patients in GINA 4/5 who all were included in the ITT population. The patients were aged 16–75 years with a clinical diagnosis of asthma for ≥6 months, severe asthma defined by requirement for high-dose ICS plus a second controller and/or systemic corticosteroids, poorly controlled asthma demonstrated by two or more severe asthma exacerbations in the preceding 12 months, requiring systemic corticosteroids plus an Asthma Control Questionnaire (ACQ)7-score > 1, and atopic status, defined as sensitisation to ≥1 perennial indoor aeroallergen [17]. All 226 patients (113 TLA / 113 placebo) with a reported baseline ACQ score were included in the current study population. Demographics for patients with ACQ7-scores reported at baseline (all and over/under 3) are presented in Table 1.

**Table 1:** Characteristics of study patients at baseline by ACQ-score (full study and score over/under 3)

	Full study^**a**^		ACQ7 > 3		ACQ7 ≤ 3	
	TLA (***n*** = 113)	Placebo (***n*** = 113)	TLA (***n*** = 42)	Placebo (***n*** = 48)	TLA (***n*** = 71)	Placebo (***n*** = 65)
Age (years)	46.7 (13.6)	44.3 (13.7)	48.6 (13.4)	42.7 (12.4)	45.6 (13.7)	45.4 (14.5)
Male sex, n (%)	33 (29)	28 (25)	14 (33)	6 (13)	19 (27)	22 (34)
BMI (kg/m^2^)	29.6 (6.0)	31.2 (7.2)	30.8 (6.0)	33.1 (6.7)	29.0 (5.9)	30.4 (7.1)
FEV_1_ (L)	2.0 (0.9)	2.0 (0.9)	1.8 (0.8)	1.7 (0.7)	2.2 (0.8)	2.3 (0.9)
FEV_1_ (% predicted)^b^	69.7 (22.6)	69.0 (21.0)	62.1 (23.9)	59.4 (19.2)	72.1 (24.2)	73.2 (24.7)
Number of exacerbations in preceding 12 months	3.2 (1.4)	3.3 (1.3)	3.5 (1.4)	3.5 (1.4)	3.1 (1.4)	3.2 (1.3)
FeNO (ppb)	32.9 (30.8)	41.8 (40.1)	35.5 (35.9)	37.7 (40.2)	31.3 (27.5)	44.9 (40.1)
Use of maintenance OCS at randomisation, n (%)	16 (15%)	24 (22%)	5 (13%)	13 (28%)	11 (16%)	11 (18%)

Values are mean (SD) unless otherwise specified

*OCS* oral corticosteroids

^a^ Numbers presented are for patients with reported ACQ7 at baseline. The total n in the LASER study was 119 vs 121

^b^ Pre-bronchodilator

### Severe asthma exacerbations

**Fig. 1:**
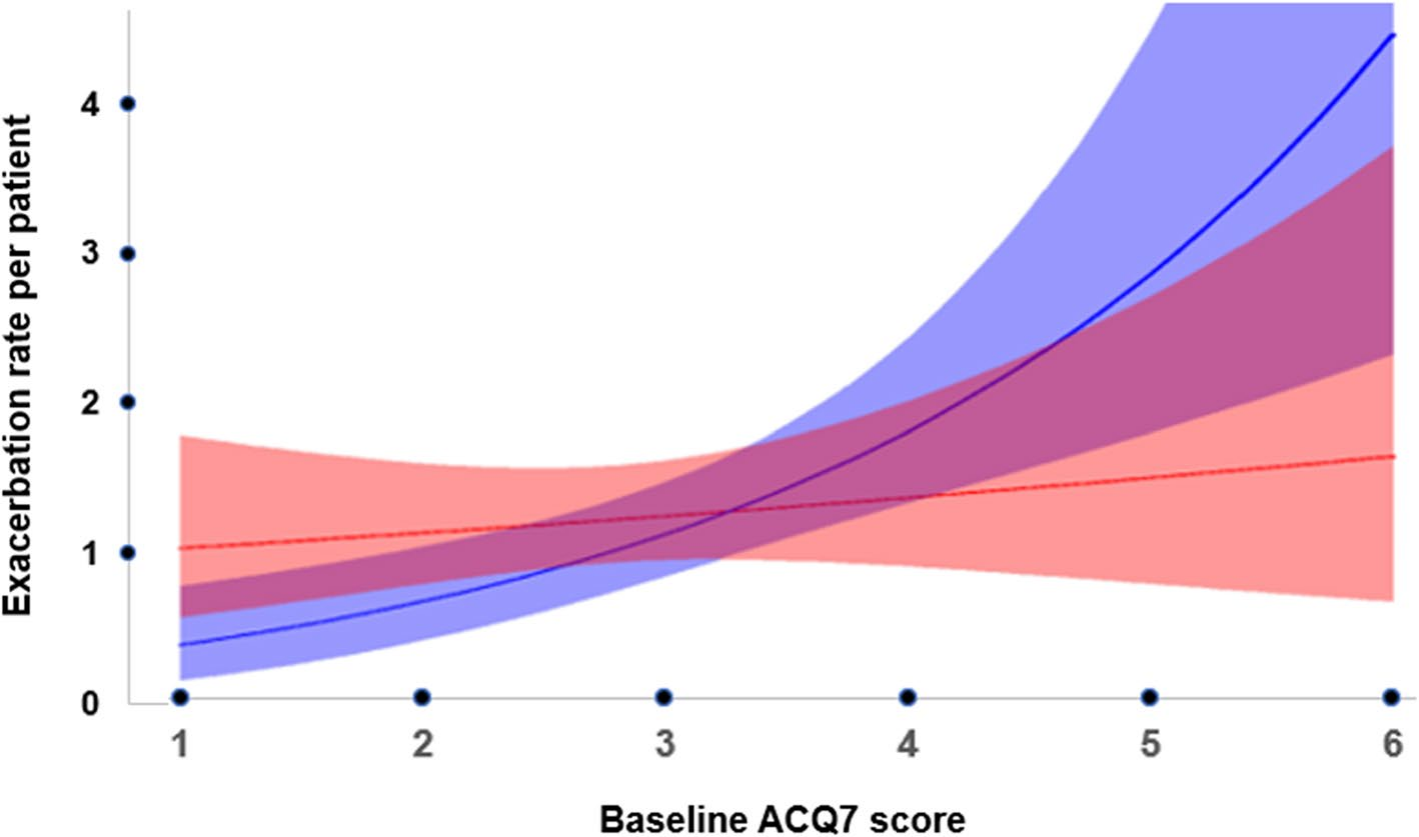
Treatment effect on severe exacerbations as a function of baseline ACQ7. (Linear interaction model with 95% CI (Negative binomial)).TLA (red) vs placebo (blue)

In the year preceding the study, patients had an exacerbation rate of around 4 exacerbations/year. During the one-year duration of the study, this rate decreased to 1.4 per year in both groups, corresponding to a 65% decrease, and 43% of the patients had zero exacerbations. Figure 1 presents the rate of severe exacerbations for TLA and placebo as a function of baseline ACQ7. In patients with more well-controlled asthma, the number of patients with no exacerbations was 35 (57%) in the placebo group compared to 23 (37%) in the TLA treatment group, contributing to overall low rates of exacerbations. For higher ACQ7 scores, a higher rate of severe exacerbation was observed and a greater separation between treatments as shown by higher rates for placebo.

**Fig. 2:**
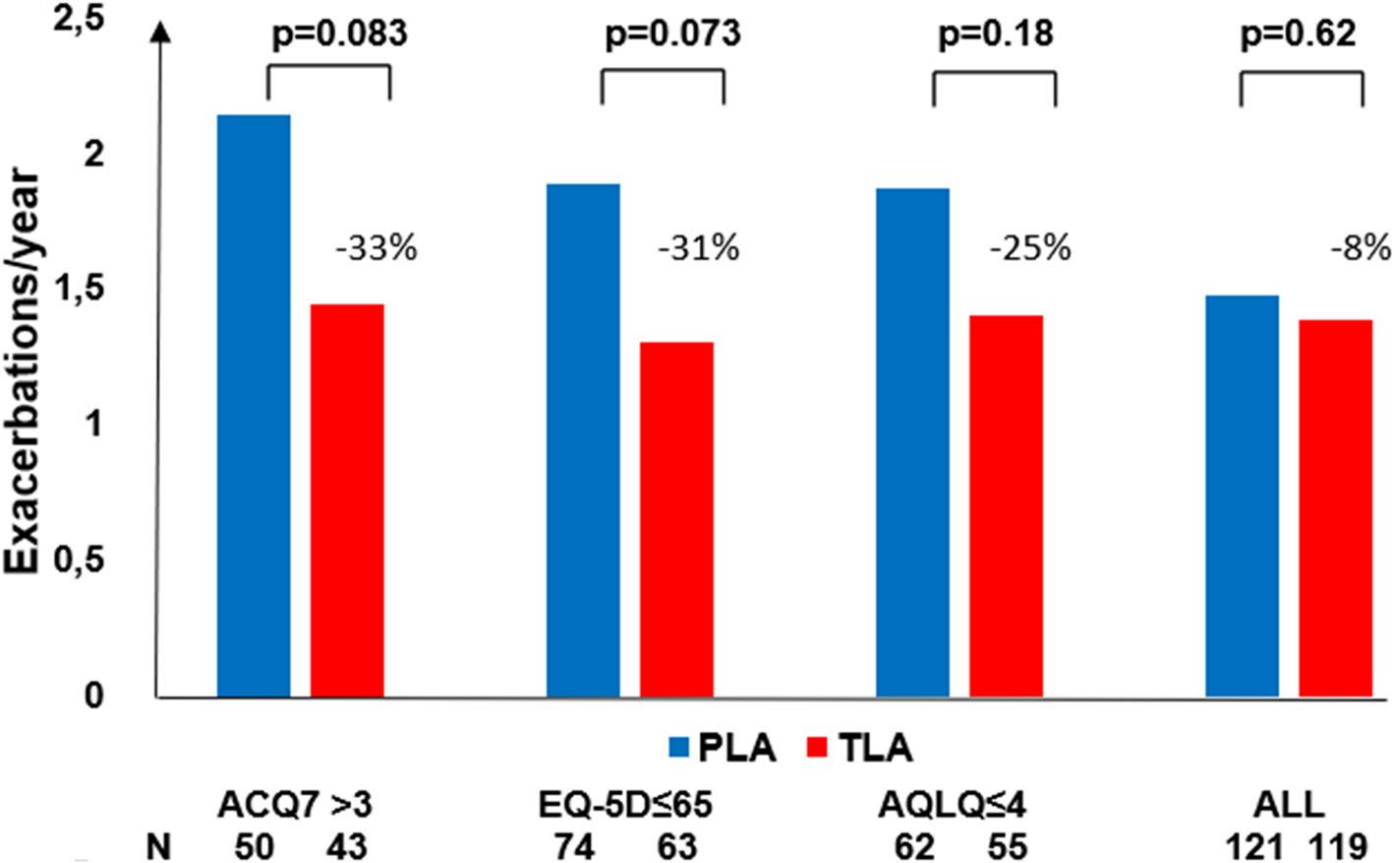
Rates of severe asthma exacerbations over 12 months for TLA (red) vs placebo (blue) for patients with ACQ7 > 3, EQ5D-VAS ≤ 65 and AQLQ≤4, and for all patients in the full study

In Table 2 and Fig. 2 the rates of severe asthma exacerbations are presented over twelve months for TLA vs placebo for patients with ACQ7 > 3, EQ5D-VAS ≤ 65 and AQLQ≤4, respectively. The rates of severe asthma exacerbations were reduced for TLA compared to placebo in the patients with more uncontrolled asthma. For patients with ACQ7 > 3 at baseline, the rate difference was 33% (*p* = 0.083); for patients with EQ5D-VAS ≤ 65, the rate difference was 31% (*p* = 0.073) and for patients with AQLQ≤4, the rate difference was 25% (*p* = 0.18). This difference should be compared to 8% (*p* = 0.62) for the full study population [17].

**Table 2:** Rate of exacerbations over 12 months by different cut-off levels for asthma control

Subgroup^a^	N TLA	N PLA	Exacerbation rate TLA	Exacerbation rate PLA	Rate ratio	***p***-value
ACQ7 > 3	43	50	1.44	2.14	0.67	0.083
EQ5D-VAS ≤ 65	63	74	1.30	1.89	0.69	0.073
AQLQ total ≤ 4	55	62	1.40	1.88	0.75	0.18

^a^ ACQ7 = 3, EQ5D-VAS = 65 and AQLQ = 4 are the median values adjusted to the nearest even integer

### Quality of life (AQLQ)

AQLQ also showed a placebo response. Fifty percent of the patients had AQLQ> 4 at randomisation which increased by 0.5 units during the first 3 months after randomisation in both groups. However, this change means a further regression to the mean, with about 50% of the patients having AQLQ> 4.5 with an average of 5.2 over the treatment period.

Table 3 presents the results for AQLQ and its domains. For patients with poorly controlled asthma (AQLQ≤4 at baseline), the difference between TLA and placebo in mean AQLQ_0-12m_ was 0.31 (*p* = 0.085), while for patients with AQLQ> 4 at baseline, no differences in mean AQLQ_0-12m_ were found (− 0.027; *p* = 0.850). A significant difference was observed in mean AQLQ_0-12m_ between TLA and placebo (0.33; *p* = 0.034), using EQ5D-VAS as baseline covariate, instead of AQLQ. With ACQ7 > 3 as the baseline covariate, the difference was 0.26 (*p* = 0.15). Table 3 also presents the improvements in the different AQLQ domains over 12 months for patients with AQLQ≤4 at baseline with AQLQ as baseline covariate. The symptom domain showed a trend, while the emotional and environmental domains showed significant differences favouring TLA. The activity domain showed a lower and non-significant difference.

**Table 3:** Mean changes in AQLQ over 12 months by different cut-off levels

Subgroup^a^	Baseline covariate	N TLA / N PLA	Mean AQLQ_**0-12m**_ TLA	Mean AQLQ_**0-12m**_ PLA	Difference	***p***-value
**AQLQ total**						
AQLQ> 4	AQLQ	60/58	5.24	5.20	−0.027	0.85
AQLQ≤4	AQLQ	55/62	4.06	3.58	0.31	0.085
AQLQ≤4	EQ5D-VAS	63/74	4.42	3.96	0.33	0.034*
AQLQ≤4	ACQ	43/50	3.89	3.38	0.26	0.15
**AQLQ domains (**AQLQ≤4)						
Symptoms	AQLQ	55/62	4.06	3.58	0.35	0.073
Activity	AQLQ	55/62	4.21	3.68	0.28	0.12
Emotional	AQLQ	55/62	4.05	3.43	0.47	0.029*
Environment	AQLQ	55/62	4.07	3.54	0.48	0.028*

^a^ ACQ7 = 3, EQ5D-VAS = 65 and AQLQ = 4 are the median values adjusted to nearest even integer

### Health economics

There was complete EQ5D-5 L utility data (at least both 12 m and baseline scores) for 85 (71%) and 93 (77%) of the TLA and placebo participants, respectively. For the TLA and placebo ACQ7 > 3 sub-groups, complete utility data were available for 30 of 43 participants (70%) and 38 of 49 participants (78%) respectively.

**Fig. 3:**
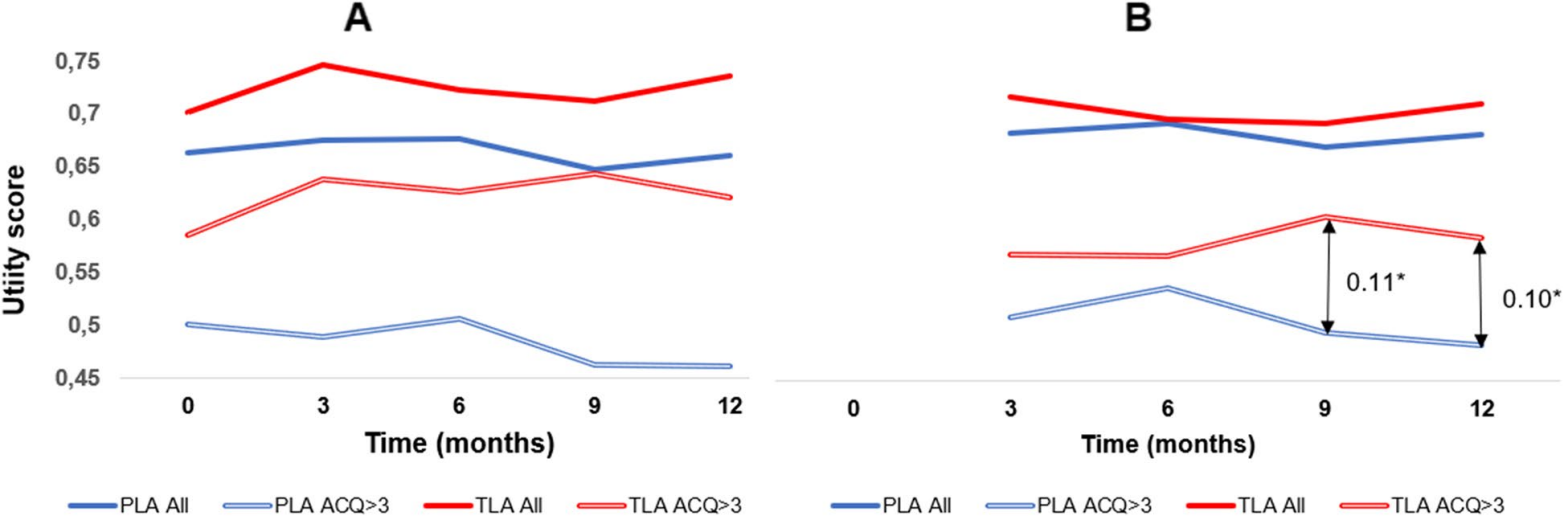
Utility scores by timepoint from the full study and the sub-group ACQ7 > 3: 3A) mean data from complete case analysis, 3B) MMRM analyses using baseline values as covariate. TLA (red) vs placebo (blue)

The sub-group defined by ACQ7 > 3 at baseline was found to have lower utility scores over the 12 months treatment period compared to the whole study in both the TLA and placebo arms (Fig. 3A), which aligns with disease severity expectations. Figure 3A also shows that the TLA and placebo groups separate over time in the ACQ7 > 3 sub-group to a greater extent than for the whole study.

The MMRM analysis was employed to adjust for missing data [18] and describe the time pattern. The differences in utility scores between TLA and placebo in the ACQ > 3 subgroup was significant at 9 and 12 months (0.11, *p* = 0.024 and 0.10, *p* = 0.046, respectively, (Fig. 3B)). The total study population’s corresponding utility values were 0.03 and 0.02 (*p* > 0.05), respectively.

**Fig. 4:**
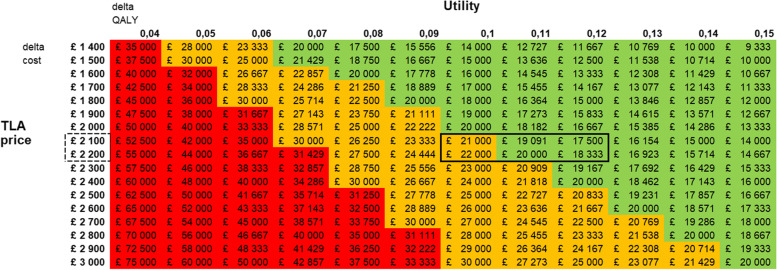
Cost-effectiveness grid over the incremental cost-effectiveness ratio (ICER) estimates for TLA from the ACQ7 > 3 sub-group. The utility scores for TLA at 9 and 12 months are shown on the Delta QALY-axis. In addition, the TLA price is shown on the Delta cost-axis resulting in cost-effectiveness (ICER) for TLA of about £17,000–£22,000 (black rectangle), which is below the acceptable recommended threshold for NICE, i.e., <£30,000

Figure 4 is a cost-effectiveness grid that shows what impact these improved treatment effects (as Quality-Adjusted Life-Year (QALY), assuming no survival gains) have on the incremental cost-effectiveness ratio (ICER) estimates for TLA. For example, where the delta cost for TLA is around £2000 to £2200, QALY gains estimated from the ACQ7 > 3 sub-group resulted in an ICER of around £20,000 (highlighted by the black rectangle in Fig. 4). This support TLA being cost effective as the acceptable recommended threshold for NICE is below £30,000 per QALY gained [12]. However, in the whole study, the utility scores were 0.02–0.03, resulting in a high ICER and no cost-effectiveness.

### Statistical considerations to the LASER study

The original power calculation was based on a Poisson regression model. The protocol stated that a treatment difference of 25%, 80% power, a significance level of 5% and a 10% drop-out rate, would require at least 222 patents (111 per group) [16]. When the original sample size was calculated, no adjustment for a possible over-dispersion was performed. A negative binomial regression model was applied the amended Final Statistical Plan.

A retrospective calculation resulted in an over-dispersion factor of 2.3 in a simple model with only treatment as a factor. With all stratification factors in the model, it was 2.2. An over-dispersion factor of 2 is in line with the results discussed in Keene et al. [19]. If this over-dispersion factor had been applied the estimated sample size would require more than twice the number of patients, i.e., > 444 patients. If the power calculation had been based on negative binomial regression (used for the analysis), using a shape parameter of 0.8, 0.9 and 1.0 the sample size had been 524, 564 and 600, respectively. A retrospective calculation resulted in a shape parameter of 0.94.

## Discussion

In this planned post hoc sub-group analysis of the LASER study [16], the more uncontrolled second half of the patient population showed consistent trends of having effects on severe exacerbations (about 30% reduction), QoL and cost-effectiveness in favour of TLA.

There could be several reasons that no effects on severe exacerbations, asthma control outcomes and health economics were demonstrated in the full LASER study - results that are inconsistent with those from the 4A study [9]. One obvious reason could be that the failure of the 12-month study designed to show effects on severe exacerbation in the recommended TLA population is a correct result, while the positive results from the other 12-month study were chance findings. However, we believe that three other reasons could have contributed to the lack of demonstrated effect in one study and not the other:

Firstly, there was a difference in the two patient populations. In the 4A study, positive results in QoL (significant) and severe exacerbations (trend) among the symptomatic GINA 4 patients could have been generated by more responsive patients in a younger population with milder disease (all GINA 5 patients on oral steroids and biologics were excluded). The LASER study included uncontrolled GINA 4/5 patients of which 24% were on oral steroids and 10% on monoclonal antibody therapy (mainly omalizumab) i.e., GINA 5. In addition, more than 50% of the patients had an FEV1/FVC < 0.7. One possible explanation for the lack of TLA effect could be that these patients were more resistant to further therapy. However, this explanation can probably be refuted, since patients in the 4A study demonstrated better effect among the more severe and symptomatic asthma patients. Our post hoc analyses of the LASER study also strengthen the assumption that the patients demonstrated a higher magnitude of difference in severe exacerbations and QoL between TLA and placebo the more uncontrolled asthma, based on both symptoms and QoL.

Secondly, the objective of the LASER study was to recruit an exacerbation-prone population (> 2 exacerbations previous year) with values of ACQ7 > 1. However, there was a large regression to the mean in that the rates of exacerbations were 65% lower in both treatment groups relative to the year preceding the study. Furthermore, 43% of the patients reported no exacerbations, with more well-controlled patients reporting no exacerbations on placebo than TLA. In addition, no difference was shown between treatments in 50% of the patients having AQLQ > 4 (average of 5.2 at randomisation). This change was further improved by 0.5 units (=MCID) 3 months after randomisation in both groups. It should also be noted that most studies with biologic therapies used an ACQ6 inclusion criterion of 1.5 (vs ACQ7 > 1 in the LASER study). Juniper et al. [20] reported validity and no loss in interpretation between ACQ6 and ACQ7, which means that the LASER study included about 15% more patients with milder symptoms than in the biologic studies.

This ‘placebo effect’ resulted in a limited chance of detecting a significant difference between TLA and placebo in the whole population. It is quite common with regression to the mean when very severe patients with strict inclusion criteria [21, 22] or when patients with poor adherence and/or poor health care access are included in clinical trials as described by Dutile et al. [23]. In their article they addressed placebo effects to pharmacological interventions but also device intervention. One speculation could be that the TLA device by the presence in the bedroom may modify and enhance the patient’s expectations of being effective. In two biologic studies [24, 25], no effect on severe exacerbations was observed, most likely because of a large placebo effect.

Thirdly, the primary variable in the LASER study was severe exacerbations and the sample size calculation did not correct for over-dispersion, which is a factor needed for a simple model such as Poisson regression. If an over-dispersion factor of 2 had been utilized [19], the study should have included twice as many patients (> 444) to balance the variance in the study. For example, exacerbation studies investigating budesonide/formoterol as maintenance and reliever therapy used an over-dispersion factor for Poisson regressions [26–29]. Today negative binomial regression is more commonly used. A sample size calculation based on this method and a shape parameter of 0.9 resulted in a sample size of 564 patients for the study. This number is similar to the above sample size calculation, provided that over-dispersion is accounted for. The shape factors of 0.8–0.9 have been used in similar patient populations, e.g., in biologic studies [30, 31]. A lower number of patients thus makes the possibility of demonstrating a favourable effect for TLA over placebo on severe exacerbations more difficult. This is also a limitation when performing our post hoc analysis in a subgroup of more severe patients from a study with low power.

In this post hoc sub-group analysis of the LASER study, we have shown a trend for the effects of TLA on severe exacerbations. The reduction of severe exacerbation rates for the three analyses performed was around 30%, which is in line with the treatment difference of 25% used in the original sample size calculations. One can only speculate if a correct 2-fold increase in sample size had given significant differences, also among the less uncontrolled patients. Reasons why TLA vs placebo failed to demonstrate an effect on severe exacerbations may include a high regression to the mean, a low power to detect a difference and a high proportion of patients with severe resistant disease on GINA 5 medication, thereby potentially having less effect or a slower onset of effect.The more symptomatic patients (defined by ACQ7 > 3) showed a similar trend effect on severe exacerbations as the patients with an ÁCT < 18 in the 4A study (*p* < 0.1). This observation may indicate that validated symptom questionnaires may be better than QoL instruments to define the right patient population to demonstrate an effect on exacerbations for TLA, as also shown by Blanco-Aparicio et al. [32]. In line with this the LASER study and the study program for biologics used ACQ as inclusion criteria in studies where severe exacerbations were the primary outcome.

In the full cost-effectiveness evaluation of the LASER study regression analysis and bootstrapping were performed to compensate for the 30% missing EQ5D-5 L utility values. We did not perform such a full cost-effectiveness analysis, i.e., our positive utility results from the LASER study are conservative. Nevertheless, the health economic analysis and the exacerbation results strengthen the documentation for TLA as being effective in patients with symptomatic severe asthma and are in line with the Scottish and Swedish recommendations [13, 14]. Comparing results between studies should always be discussed with care. That said the effect of TLA in the more severe asthma patients seems to be somewhat lower for exacerbations (30% versus about 50% for biologics), while the effect on QoL (0.30 score unit) and FEV_1_ (limited effect) are similar to biologics [3, 4]. The cost-effectiveness is, however, in favour of TLA with an ICER per QALY of <£30,000, while no biologics has achieved an ICER per QALY below $150,000 [3–5].

Our results are consistent with those from a recent meta-analysis [33] where the more symptomatic patients with severe allergic asthma from the 4A study [9] (GINA 4/5 only; *n* = 129) and the LASER study (all patients; *n* = 235) were combined. In this study, TLA was shown to have a significant effect compared to placebo in patients with baseline ACQ > 3 or ACT< 18 for severe exacerbations (41% reduction, *p* = 0.015) and AQLQ responders (74% vs 43%, *p* = 0.04), respectively. Note that health economics was not collected in the 4A study and hence not included in the meta-analysis.

A limitation of this study is the post-hoc nature of the analysis. The objective of investigating effects on more uncontrolled severe asthma was already outlined in the 4A study, where the prespecified secondary analysis should include more symptomatic and severe patients, defined as ACT< 18 and GINA 4. In addition, the LASER protocol included further explorative analysis around severity. Another limitation is related to the number of patients, which was too low for the subgroup analyses – potentiated by the full study having low power, as discussed in the statistical section. Nevertheless, the explorative analyses showed trends for severe exacerbations and AQLQ, significance for some subdomains of AQLQ and cost-effectiveness. This leads to another limitation, which includes multiple analyses. This is factual, but the exploration also indicates consistency in the LASER results and consistency between LASER and the 4A study for severe exacerbations and quality of life when patients with more symptomatic severe asthma are studied. It could also be argued that TLA only affected the most uncontrolled patients with severe asthma. This is our finding, but it should also be recognised that the significant effect on severe exacerbations depend on effect size and the number of patients i.e., few patients demand a big effect difference. The results challenge the full LASER results but the insight from the full LASER study and this post hoc analysis should be addressed in a new study.

## Conclusion

In conclusion, our sub-group analysis demonstrated a difference in the effect of TLA over placebo on severe exacerbations, asthma control and health economics in symptomatic severe asthma. Unfortunately, this improvement was not demonstrated in the full study. Several explanations for the different outcomes have been outlined and should be addressed in a new study. However, these results align with the documentation of TLA as being effective and cost-effective in patients with symptomatic severe asthma, the patient population for which TLA is intended.

## Data Availability

The datasets used and/or analysed during the current study are available from the corresponding author on reasonable request.

## Abbreviations

TLA: Temperature-controlled Laminar Airflow
ACQ: Asthma Control Questionnaire
AQLQ: Asthma Quality of Life Questionnaire
EQ5D-5L: EuroQoL 5-Dimension 5-Level Questionnaire
EQ5D-VAS: EuroQoL 5-Dimension Visual Analogue Scale
GINA: Global Initiative for Asthma
ICS: Inhaled corticosteroid
RCT: Randomised controlled trial
FeNO: Fraction exhaled Nitric Oxide
ICER: Incremental Cost-Effectiveness Ratio
LASER: Laminar Airflow in Severe Asthma for Exacerbation Reduction (trial)
MCID: Minimal Clinically Important Difference
MMRM: Mixed effect Model Repeat Measurement
NICE: National Institute for Health and Care Excellence
PLA: Placebo
QALY: Quality-Adjusted Life-Year
